# A Novel Detection Method for Underwater Moving Targets by Measuring Their ELF Emissions with Inductive Sensors

**DOI:** 10.3390/s17081734

**Published:** 2017-07-28

**Authors:** Jinhong Wang, Bin Li, Lianping Chen, Li Li

**Affiliations:** 1 The Key Laboratory of Ocean Acoustics and Sensing, Ministry of Industry and Information Technology, School of Marine Science and Technology, Northwestern Polytechnical University, Xi’an 710072, China; libin cme@nwpu.edu.cn (B.L.); chenlianping@nwpu.edu.cn (L.C.); 2 The Institute of Electronic Engineering, China Academy of Engineering Physics, Mianyang 621900, China; lili@mtrc.ac.cn

**Keywords:** underwater targets, ELF emissions, horizontal electric dipole, three-layer media, inductive sensors

## Abstract

In this article, we propose a novel detection method for underwater moving targets by detecting their extremely low frequency (ELF) emissions with inductive sensors. The ELF field source of the targets is modeled by a horizontal electric dipole at distances more than several times of the targets’ length. The formulas for the fields produced in air are derived with a three-layer model (air, seawater and seafloor) and are evaluated with a complementary numerical integration technique. A proof of concept measurement is presented. The ELF emissions from a surface ship were detected by inductive electronic and magnetic sensors as the ship was leaving a harbor. ELF signals are of substantial strength and have typical characteristic of harmonic line spectrum, and the fundamental frequency has a direct relationship with the ship’s speed. Due to the high sensitivity and low noise level of our sensors, it is capable of resolving weak ELF signals at long distance. In our experiment, a detection distance of 1300 m from the surface ship above the sea surface was realized, which shows that this method would be an appealing complement to the usual acoustic detection and magnetic anomaly detection capability.

## 1. Introduction

With the rapid development of ocean exploitation and military applications around the world, there has been a significant rise in the requirement for the measurement, surveillance and warning of the ship’s physical fields. With relatively longer distance transmission capability, acoustics has become a proven technology for detection of the underwater moving targets. However, the substantial improvement of the acoustics stealth technique and increasingly adverse acoustic environments in practical applications have raised serious concerns on the acoustic detection method. On the other hand, with the increasing enhancement of the performance of electric/magnetic sensors, many countries have devoted considerable effort to the study of the electromagnetic field sources of the underwater targets, such as submarine, unmanned underwater vehicle (UUV), etc., and their relevant detection problem [1,2,3]. Among these sources, ferromagnetic field source has been studied thoroughly and widely applied in magnetic anomaly detection (MAD) systems. However, the strength of this source can be largely minimized by using degaussing technology, and MAD signals usually suffer from strong magnetic noise interference from geology, geomagnetic, platform vibration and motion, ocean motion and wave, which severely limit the application of MAD systems in realistic environments [2].

In recent years, the corrosion related shaft-rate electromagnetic field source of the underwater moving targets has generated great interest. It produces extremely low frequency (ELF) emissions, which arise from the modulation of the corrosion currents of the targets and are expected to be of substantial value in poorly maintained platforms [4,5,6,7,8,9]. These emissions offer unique advantages over MAD signals as follows: first, at ELF detection frequencies, the usual electromagnetic noise interference should be much less of an issue than in the MAD frequency band; second, ELF emissions have relative low propagation attenuation which can be detected at long distances or deep locations in seawater.

In this article, a novel detection method for underwater moving targets by utilizing their ELF emissions is proposed. The ELF field source is modeled by a horizontal electric dipole (HED) submerged in seawater at long distances. The formulas for electromagnetic fields in air produced by the HED are derived and computed with a complementary numerical integration technique. Finally, a detection experiment of a surface ship on shallow sea is presented, inductive sensors were employed to collect the ELF signals emitted from the ship in the experiment. The test data verify the effectiveness of the proposed method. This article mainly focuses on the issues of modeling the ELF field source and the propagation of the ELF waves in shallow sea environment. The methods of signal processing and noise rejection will be considered in the next phase work.

## 2. Theoretical Modeling and Analysis

Since the ELF emissions are generated by the periodic modulation of the corrosion currents of the targets with their propellers’ rotating, we assume the ELF source can be modeled by a HED with electric moment of Il at distances longer than several times of the target length, where *I* is the current in the shaft after modulation, and *l* is approximately equal to the distance from the propeller to the auxiliary anode. Base on this assumption, the ELF emissions produced in air by underwater targets can be evaluated. Although the propagation of waves through multilayered medium has been extensively studied [10,11,12,13,14,15,16], there has been comparatively little effort to determine the fields produced in air by a HED submerged in a shallow sea. We will derive the field expressions with recursive propagation approach described in [14].

We first consider a HED embedded in an unbounded homogeneous medium, it is directed in the *x* direction, i.e., $J=\hat{x}Il\delta \left(r\right)$. Then its electric and magnetic fields can be derived easily via the Green’s function approach as

(1) $$\begin{array}{ccc} E(r)\;\;\; & = & \;\;\;-j\omega \mu \left(\bar{I}+\frac{\nabla \nabla }{k^{2}}\right)\cdot \hat{x}Il\frac{e^{-jkr}}{4\pi r} \end{array}$$

(2) $$\begin{array}{ccc} H(r)\;\;\; & = & \;\;\;\nabla \times \hat{x}Il\frac{e^{-jkr}}{4\pi r} \end{array}$$

where $k=\omega \sqrt{\mu \epsilon }$ is the wave number of the medium. With the well-known Sommerfeld identity, the transverse magnetic (TM) and transverse electric (TE) fields can be derived easily from Equations (1) and (2) as

(3) $$\begin{array}{ccc} E_{z}\;\;\; & = & \;\;\;\mp \frac{jIl}{4\pi \omega \epsilon }\cos\phi \int_{0}^{\infty }dk_{\rho }k_{\rho }^{2}J_{1}\left(k_{\rho }\rho \right)e^{-jk_{z}\left|z\right|} \end{array}$$

(4) $$\begin{array}{ccc} H_{z}\;\;\; & = & \;\;\;-\frac{jIl}{4\pi }\sin\phi \int_{0}^{\infty }dk_{\rho }\frac{k_{\rho }^{2}}{k_{z}}J_{1}\left(k_{\rho }\rho \right)e^{-jk_{z}\left|z\right|} \end{array}$$

where $k_{z}=\sqrt{k^{2}-k_{\rho }^{2}}$. Since $E_{z}$ is odd about the source, the downgoing wave has an opposite sign from the upgoing wave.

**Figure 1:**
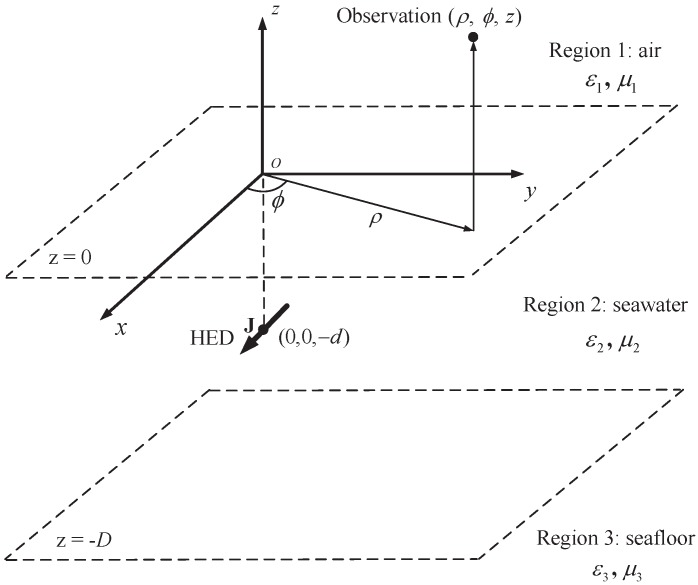
A horizontal electric dipole in three-layer media.

Now we consider a three-layer medium consisting of air (region 1: z≥0), seawater with depth of *D* (region 2: −D≤z<0) and seafloor (region 3: z<−D), as illustrated in Figure 1. The fields generated in air by a HED submerged in seawater with depth of *d* are desired. The *i*-th (i=1,2,3) layer is characterized by permeability $\mu_{i}$ and permittivity $\epsilon_{i}$, where $\mu_{i}=\mu_{0}$ and $\epsilon_{i}=\epsilon_{0}\epsilon_{ri}-j\sigma_{i}/\omega$. $\epsilon_{0}$ and $\mu_{0}$ represent the permittivity and permeability of free space, respectively. z=0 and z=−D are the positions of the boundaries.

Then Equations (3) and (4) multiplied by the amplitude $A_{1}$ represent the fields in air generated by the source. $A_{1}$ can be related to the upgoing wave amplitude $A_{2}$ in seawater at z=0 as

(5) $$A_{1}^{TE/TM}=A_{2}^{TE/TM}T_{21}^{TE/TM}$$

for TE and TM waves, $A_{2}$ is given as

(6) $$\begin{array}{ccc} A_{2}^{TE}\;\; & = & \;\;\frac{e^{-jk_{2z}d}+e^{-jk_{2z}\left(2D-d\right)}R_{23}^{TE}}{1-R_{21}^{TE}R_{23}^{TE}e^{-2jk_{2z}D}} \end{array}$$

(7) $$\begin{array}{ccc} A_{2}^{TM}\;\; & = & \;\;\frac{e^{-jk_{2z}d}-e^{-jk_{2z}\left(2D-d\right)}R_{23}^{TM}}{1-R_{21}^{TM}R_{23}^{TM}e^{-2jk_{2z}D}} \end{array}$$

The poles of the denominators imply the guidance condition (also known as the transverse resonance condition) for guided modes in seawater. The number of guided-mode poles depends on the frequency and the seawater depth, higher frequency and larger depth give rise to more guided-mode poles.

In Equations (5)–(7), $R_{ij}$ and $T_{ij}$ are the Fresnel reflection and transmission coefficients, for TE and TM waves, they are

(8) $$\begin{array}{ccc} R_{ij}^{TE}\;\;\; & = & \;\;\;\frac{\mu_{j}k_{iz}-\mu_{i}k_{jz}}{\mu_{j}k_{iz}+\mu_{i}k_{jz}};\;\;\;T_{ij}^{TE}=\frac{2\mu_{j}k_{iz}}{\mu_{j}k_{iz}+\mu_{i}k_{jz}} \end{array}$$

(9) $$\begin{array}{ccc} R_{ij}^{TM}\;\;\; & = & \;\;\;\frac{\epsilon_{j}k_{iz}-\epsilon_{i}k_{jz}}{\epsilon_{j}k_{iz}+\epsilon_{i}k_{jz}};\;\;T_{ij}^{TM}=\frac{2\epsilon_{j}k_{iz}}{\epsilon_{j}k_{iz}+\epsilon_{i}k_{jz}} \end{array}$$

Applying Equations (3)–(5) and the boundary conditions at z=0, we can obtain the vertical fields of $E_{1z}$ and $H_{1z}$. The integrands of the transverse fields of $E_{1\rho }$, $E_{1\phi }$, $H_{1\rho }$ and $H_{1\phi }$ can be found from

(10) $$\begin{array}{ccc} {\tilde{E}}_{t}\;\;\;\; & = & \;\;\;\;\frac{1}{k_{\rho }^{2}}\left[\nabla_{s}\frac{\partial {\tilde{E}}_{z}}{\partial z}+j\omega \mu \hat{z}\times \nabla_{s}{\tilde{H}}_{z}\right] \end{array}$$

(11) $$\begin{array}{ccc} {\tilde{H}}_{t}\;\;\;\; & = & \;\;\;\;\frac{1}{k_{\rho }^{2}}\left[\nabla_{s}\frac{\partial {\tilde{H}}_{z}}{\partial z}-j\omega \epsilon \hat{z}\times \nabla_{s}{\tilde{E}}_{z}\right] \end{array}$$

where $\nabla_{s}=\nabla -\hat{z}\frac{\partial }{\partial z}$. Then the expressions for ${\tilde{E}}_{t}$ and ${\tilde{H}}_{t}$ can be integrated to obtain the transverse fields. All the six field components are given as

(12) $$\begin{array}{ccc} E_{1z}\;\;\;\; & = & \;\;\;\;-\frac{jIl}{4\pi \omega \epsilon_{1}}\cos\phi \int_{0}^{\infty }dk_{\rho }k_{\rho }^{2}J_{1}\left(k_{\rho }\rho \right)A_{1}^{TM}e^{-jk_{1z}z} \end{array}$$

(13) $$\begin{array}{ccc} H_{1z}\;\;\;\; & = & \;\;\;\;-\frac{jIl}{4\pi }\sin\phi \int_{0}^{\infty }dk_{\rho }\frac{k_{\rho }^{2}}{k_{2z}}J_{1}\left(k_{\rho }\rho \right)A_{1}^{TE}e^{-jk_{1z}z}\; \end{array}$$

(14) $$\begin{array}{ccc} E_{1\rho }\;\;\;\; & = & \;\;\;\;-\frac{Il}{4\pi \omega \epsilon_{1}}\cos\phi \int_{0}^{\infty }dk_{\rho }k_{1z}J_{1}^{\prime }\left(k_{\rho }\rho \right)A_{1}^{TM}e^{-jk_{1z}z}\;\;\\ & & \;\;\;\;-\frac{Ilk_{1}^{2}}{4\pi \omega \epsilon_{1}\rho }\cos\phi \int_{0}^{\infty }\frac{dk_{\rho }}{k_{2z}}J_{1}\left(k_{\rho }\rho \right)A_{1}^{TE}e^{-jk_{1z}z} \end{array}$$

(15) $$\begin{array}{ccc} E_{1\phi }\;\;\;\; & = & \;\;\;\;\frac{Il}{4\pi \omega \epsilon_{1}\rho }\sin\phi \int_{0}^{\infty }dk_{\rho }k_{1z}J_{1}\left(k_{\rho }\rho \right)A_{1}^{TM}e^{-jk_{1z}z}\;\;\\ & & \;\;\;\;+\frac{Ilk_{1}^{2}}{4\pi \omega \epsilon_{1}}\sin\phi \int_{0}^{\infty }\frac{dk_{\rho }}{k_{2z}}J_{1}^{\prime }\left(k_{\rho }\rho \right)A_{1}^{TE}e^{-jk_{1z}z} \end{array}$$

(16) $$\begin{array}{ccc} H_{1\rho }\;\;\;\; & = & \;\;\;\;-\frac{Il}{4\pi \rho }\sin\phi \int_{0}^{\infty }dk_{\rho }J_{1}\left(k_{\rho }\rho \right)A_{1}^{TM}e^{-jk_{1z}z}\;\;\\ & & \;\;\;\;-\frac{Il}{4\pi }\sin\phi \int_{0}^{\infty }dk_{\rho }\frac{k_{1z}}{k_{2z}}J_{1}^{\prime }\left(k_{\rho }\rho \right)A_{1}^{TE}e^{-jk_{1z}z} \end{array}$$

(17) $$\begin{array}{ccc} H_{1\phi }\;\;\;\; & = & \;\;\;\;-\frac{Il}{4\pi }\cos\phi \int_{0}^{\infty }dk_{\rho }J_{1}^{\prime }\left(k_{\rho }\rho \right)A_{1}^{TM}e^{-jk_{1z}z}\;\;\\ & & \;\;\;\;-\frac{Il}{4\pi \rho }\cos\phi \int_{0}^{\infty }dk_{\rho }\frac{k_{1z}}{k_{2z}}J_{1}\left(k_{\rho }\rho \right)A_{1}^{TE}e^{-jk_{1z}z} \end{array}$$

where $J_{1}^{\prime }\left(k_{\rho }\rho \right)=k_{\rho }J_{0}\left(k_{\rho }\rho \right)-J_{1}\left(k_{\rho }\rho \right)/\rho$. Then the ELF emissions in air from the underwater targets can be evaluated.

## 3. Calculation and Experiment

### 3.1. Numerical Integration Technique

The calculation of the fields requires the evaluation of the Sommerfeld integrals (SIs). A large number of techniques have been developed to evaluate the SIs with both analytical and numerical methods. However, none of these analytical-numerical techniques are valid for general source orientation and observation location or arbitrary medium parameters. As a compromise between accuracy and efficiency, we evaluate the SIs utilizing the method described in [17]. This method combines two different numeral integration techniques in a complementary manner. The first one is Gauss-Laguerre quadrature, which works very well for ρ/ξ<1 (ξ is the vertical distance from the source). The second integration technique is called the Romberg-Shanks composite method, which has been found effective for ρ/ξ≥1.

The Romberg-Shanks integration is performed between the zeros of the Bessel function and the truncated infinite integral is expressed as a sum of integral between successive Bessel zeros. Since the summation usually converges very slowly due to the rapidly oscillating Bessel function, especially for large values of argument, Shanks transform are used to accelerate the convergence [18]. The integrals between Bessel zeros can be efficiently evaluated with Romberg adaptive quadrature.

### 3.2. Test Environment Description

**Figure 2:**
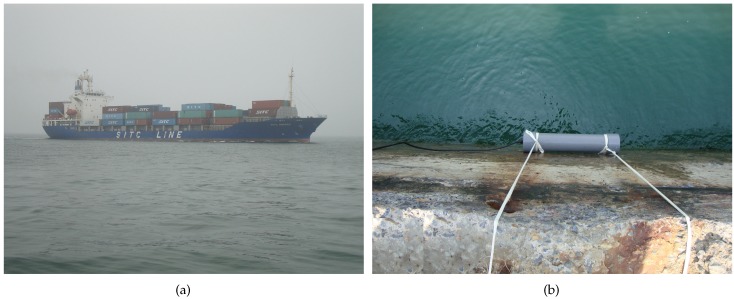
The detection experiment on a shallow sea. (**a**) the surface ship as the target; (**b**) the magnetic field sensor above the sea surface.

A detection experiment was conducted on a shallow sea to provide an experimental basis for the validation of the proposed detection method. The experimental location was chosen near the coast of Dalian, Liaoning Province of China. The seawater depth is in the range of 15∼20 m. For convenience, we adopted a surface ship as the target, as shown in Figure 2a. The fields were measured with electric and magnetic sensors as the ship was leaving the harbor. An inductive magnetic sensor with high sensitivity of 216 mV/nT and low noise level of 0.057 pT/$\sqrt{\mathrm{Hz}}$ at 1 Hz was placed 0.5 m above the sea surface against the wall of a dam, as shown in Figure 2b. The description of this sensor can be found in Appendix A. For the electric sensor we used the Ag/AgCl electrodes and integrated ultra low-noise pre-amplifiers, which is self-developed based on the N-channel silicon junction field-effect transistor (JFET) of IF 9030. This sensor has low noise less than 1 nV/$\sqrt{\mathrm{Hz}}$ at 1 Hz and was placed beside the ship’s course, which was monitoring by a a laser range finder. Due to the high performance of these sensors, it is capable of detecting the target’s ELF signals at long distances. A Cartesian coordinate was built for reference by setting the sensor’ position as the origin, setting the ship’s moving direction as the *x* direction and its abeam direction as the *y* direction.

### 3.3. Experimental Results and Discussions

**Figure 3:**
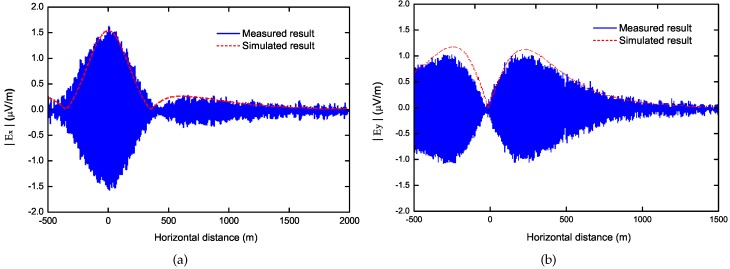
Comparisons of the simulated and measured electric field strength. (**a**) $|E_{x}|$; (**b**) $|E_{y}|$.

In last section, we assumed that the ELF field source of the underwater moving targets could be modeled as a HED submerged in sea. To prove this assumption, we measured the variation of electric field strength both in the *x* and *y* directions as the ship was leaving the harbor, and the test results were compared with the simulated results produced by an *x*-directed HED in seawater with depth of 2 m below the sea surface in Figure 3. The depth of the HED dipole is determined by the depth of the ship’s propeller and auxiliary anode, which is affected by the ship’s structure, weight, carrying capacity, etc.Basically, we adapt all the simulation conditions corresponding to the experiment conditions. They are summarized in Table 1, in which the parameters are referred to Figure 1. A good agreement of them demonstrates our assumption. The equivalent electric moment is about 40 A·m, which is evaluated with the corrosion current and parameters of the ship’s structure and material parameters, including the distance between propeller and auxiliary anode, conductivity and surface area of the shell, length and conductivity of the shaft, surface area of the propeller, etc. In many cases, the equivalent electric moment can also be evaluated with the measured data of the electric/magnetic fields around the ship.

**Figure 4:**
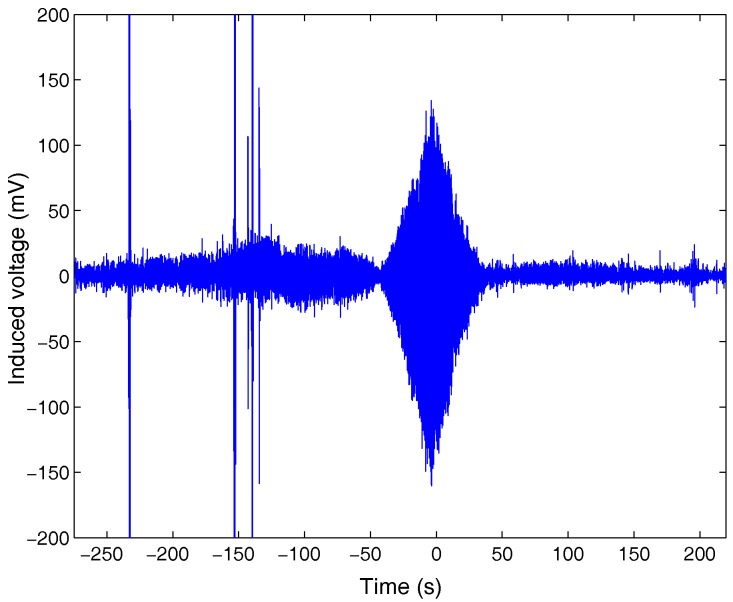
The original voltage signal induced on magnetic sensor.

**Figure 5:**
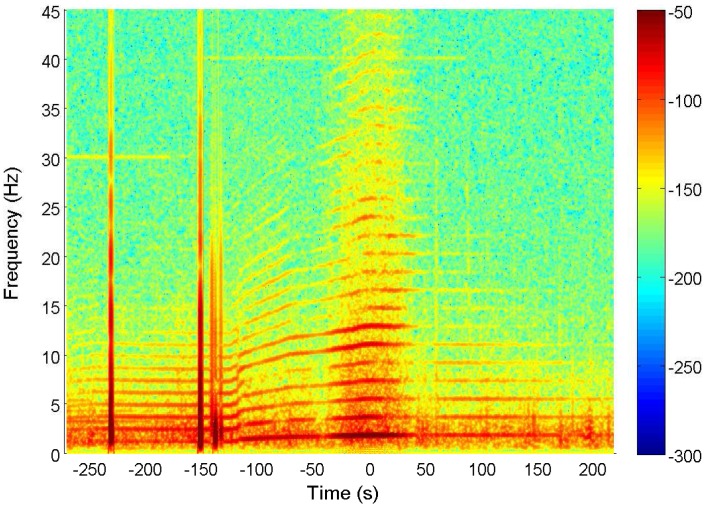
The time-frequency characteristic of the magnetic strength.

Based on the above conclusion, we begin to analyze the ship’s ELF emissions with the test data of the magnetic field. Figure 4 provides the original voltage signal induced on the magnetic sensor. There are several sharp burrs around −250 s and −150 s, which are introduced by the vibration of the magnetic sensor during the test. After performing a fast Fourier transform (FFT) to the received signal, we obtain the time-frequency characteristic of the magnetic field, as shown in Figure 5. The line spectrums in the figure represent the fundamental frequency and harmonic frequencies in ELF band. The density of the line spectrums varies from dense to sparse, and then becomes steady after passing the abeam direction. Actually, this process describes the variation of the ship’s speed. As inside the harbor, the speed was slow, i.e., the propeller rotated slowly which produced low fundamental frequency. The harmonic frequencies were integral multiple of the fundamental frequency, which led to the dense line spectrums. Similarly, the ship’s high speed outside the harbor led to the sparse line spectrums. From the figure, we can see the ship began to accelerate at −100 s, and tended to sail steadily after zero.

**Figure 6:**
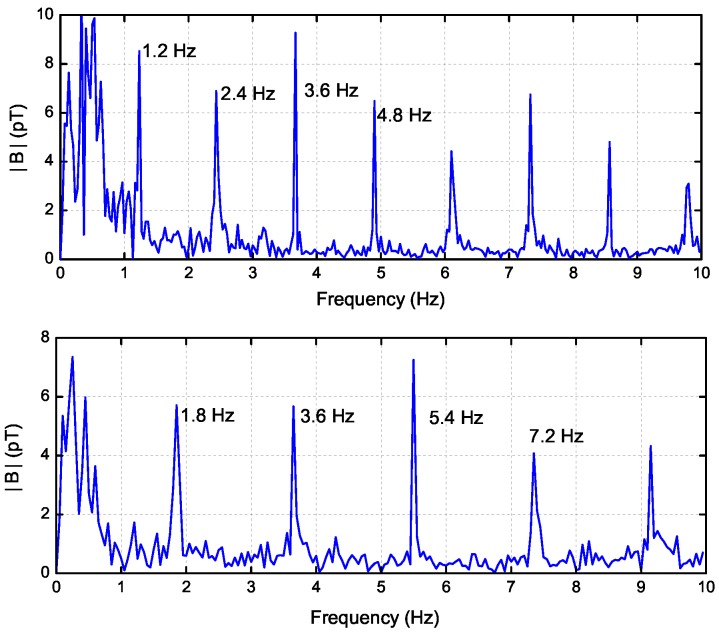
The spectrum signatures before (**upper**) and after (**lower**) the target’s acceleration.

In order to clearly show the relationship between the speed and the frequency of ELF emissions, we compare the spectrum signatures before and after the ship’s acceleration in Figure 6. Before acceleration, the fundamental and harmonic frequencies are 1.2 Hz, 2.4 Hz, 3.6 Hz, etc., after acceleration, they become as 1.8 Hz, 3.6 Hz, 5.4 Hz, etc. This characteristic obviously shows the relationship. These harmonic line spectra are of great value for the identification of the underwater moving targets.

**Figure 7:**
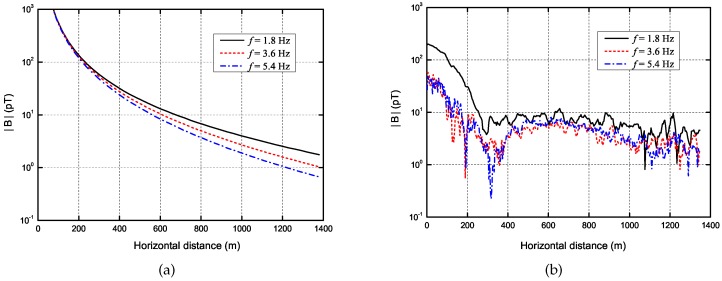
Attenuation of the magnetic strength with ρ at 1.8 Hz, 3.6 Hz and 5.4 Hz. (**a**) Simulation; (**b**) Measurement.

Now we analyze the propagation attenuation of the ship’s ELF emissions. Although a number of harmonic frequencies were generated, only the fundamental frequency of 1.8 Hz and the first two harmonic frequencies of 3.6 Hz and 5.4 Hz are considered for simplicity. Figure 7 shows the simulated and measured propagation attenuation at these frequencies. The simulation conditions can be found in Table 1. The measured results agree well with simulated results at distances more than 500 m. The divergence is significant at distances less than 500 m due to the interferences of the ship’s ferromagnetic signal and the magnetic noise introduced by the ship’s motion. Both simulated and measured results show that the magnetic field strength at fundamental frequency is the strongest, and the attenuation become higher with increasing frequency. In addition, the magnetic strength decays sharply at very short distances less than 300 m and decays relatively slowly at distances more than 300 m, which is caused by the effect of the sea surface.

**Figure 8:**
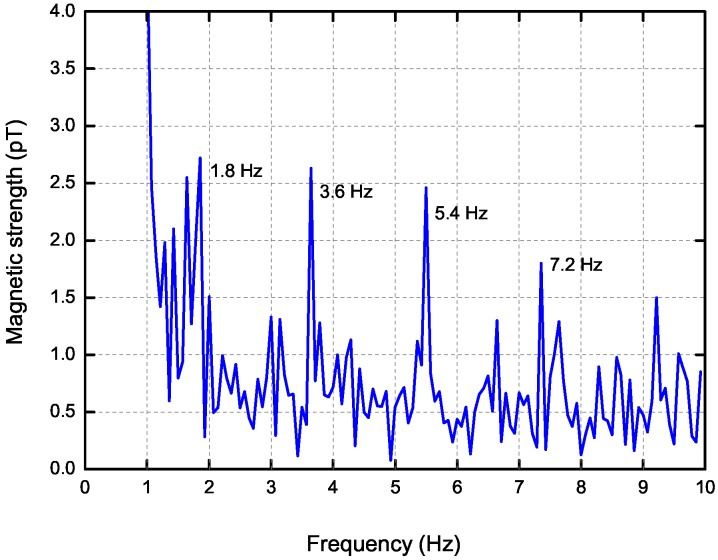
The measured magnetic strength at distance of 1300 m.

In order to show that the target can be detected at long distance with its ELF emissions, Figure 8 presents the measured magnetic strength at long distance of 1300 m. Typical line spectrums are observed, and the magnetic strength still have substantial values over 2 pT at 1.8 Hz, 3.6 Hz and 5.4 Hz. During the test, the detection distance could be much longer than than 1300 m if we did not stop the experiment and employed powerful signal processing algorithms.

## 4. Conclusion

A novel detection method for underwater moving targets with their ELF electromagnetic emissions has been proposed in this article. The ELF field source is modeled by an HED submerged in seawater. The radiation from the source on the basis of a three-layer model is evaluated. A proof of concept measurement was conducted on shallow sea utilizing a surface ship as the target. The measured results prove the following conclusions: first, the ELF field source of the target can be modeled by a HED; second, harmonic line spectrums are produced by the ELF field source, and there is a direct relationship between the ship’s speed and the frequency of the line spectrums; finally, longer detection distance can be achieved with the proposed detection method as compared with the traditional MAD method. A detection distance of 1300 m for a surface ship was realized with the help of the inductive sensors of high performance. This exhibits the great potential of the proposed method in many practical applications of underwater moving targets detection based on the platforms of aerial vehicles, air-dropped buoys and monitoring stations.

## Figures and Tables

**Table 1:** The simulation conditions corresponding to the experiment conditions.

Parameters	Values
Dipole Direction	*x*-directed
Current Moment (*Il*)	40 A·m
Frequencies (*f*)	1.8 Hz, 3.6 Hz, 5.4 Hz
Dipole Depth (*d*)	∼2 m
Horizontal Distances (ρ)	0∼2000 m
Azimuthal Angle (ϕ)	∼$\pm 90^{o}$ (i.e., the *y*-direction)
Receiver Heights (*z*)	magnetic sensor at z=0.5 m
	electric sensor at z=−0.1 m
Boundary Positions	sea surface at z=0 m
	seafloor interface at z=−D=−17.5 m (average)
Relative Permittivity ($\epsilon_{ri}$)	$\epsilon_{r1}=1$, $\epsilon_{r2}=80$, $\epsilon_{r3}=10$
Permeability ($\mu_{i}$)	$\mu_{1}=\mu_{2}=\mu_{3}=\mu_{0}=4\pi \times 10^{-7}$ H/m
Conductivity $\sigma_{i}$	$\sigma_{1}=0$ S/m, $\sigma_{2}=4$ S/m, $\sigma_{3}=0.01$ S/m

**Figure A1:**
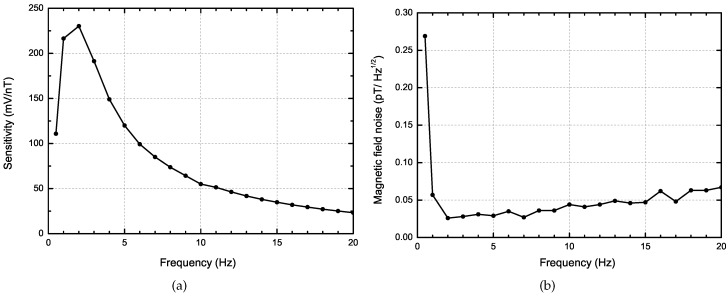
The key performance parameters of the inductive magnetic sensor versus the frequency. (**a**) Sensitivity; (**b**) Spectral density of the magnetic field noise.

## Abbreviations

The following abbreviations are used in this manuscript:

ELF	extremely low frequency
UUV	unmanned underwater vehicle
MAD	magnetic anomaly detection
HED	horizontal electric dipole
TM	transverse magnetic
TE	transverse electric
SIs	Sommerfeld integrals
JFET	junction field-effect transistor
FFT	fast Fourier transform

## Appendix A

The inductive magnetic sensor used in the experiment is self-developed by the Xi’an Huashun Measuring Equipment Company (Xi’an, China), which is composed of induction coils, magnetic core, amplifier and feedback circuits. It works on the well-known principle of Faraday’s law of induction. Its main advantages are a breakthrough in the bottleneck for improvement of sensitivity and magnetic field noise level with such a compact size (diameter of 44 mm and length of 470 mm). The responding band width is from 0.5 Hz to 20 Hz. It can measure weak magnetic fields with high accuracy and resolution, which help us detect the target’s weak ELF signals at long distances in the experiment. The sensitivity and magnetic field noise spectral density of the inductive magnetic sensor versus the frequency are provided in Figure A1. These data were tested in a magnetically shielded room. Its main technical parameters are listed in Table A1.

**Table A1:** The main technical parameters of the inductive magnetic sensor.

Parameter	Values
Measuring Range	±10 nT
Band Width	0.5∼20 Hz
Sensitivity	216 mV/nT at 1 Hz
Spectral Noise	0.057 pT at 1 Hz
Working Voltage	±3 V∼±5 V
Power Consumption	3∼5 mW
Product Size	Ø 44 mm × 470 mm
Weight	<1 kg
